# Spider Assemblages of Tree Trunks and Tree Branches in Three Developmental Phases of Primeval Oak–Lime–Hornbeam Forest in the Białowieża National Park

**DOI:** 10.3390/insects13121115

**Published:** 2022-12-03

**Authors:** Marzena Stańska, Tomasz Stański

**Affiliations:** 1 Institute of Biological Sciences, Faculty of Sciences, Siedlce University of Natural Sciences and Humanities, 08-110 Siedlce, Poland; 2 Faculty of Sciences, Siedlce University of Natural Sciences and Humanities, 08-110 Siedlce, Poland

**Keywords:** arboreal spiders, the Białowieża Forest, primeval forest

## Abstract

**Simple Summary:**

At present, the only place in Europe where the full development cycle of forests takes place on a large scale is the Białowieża Forest, because in most other forests dead or dying trees are eliminated, so the terminal (decay) phase does not occur there. Studies of animal assemblages inhabiting different forest phases are scarce as well as studies of spiders inhabiting tree trunks and branches. In this study, we compare spider assemblages inhabiting the tree trunks and branches in the optimal, terminal and regeneration phases of a primeval oak–lime–hornbeam stand in terms of their abundance, species diversity and species richness. We did not find differences in the total spider species richness between the analysed phases. However, we found that species diversity of both foliage-dwelling and trunk-dwelling spider assemblages was higher in the terminal phase compared to the other phases, which may indicate that this phase offers the most diverse niches for spiders as a result of the significant disturbance in the forest stand structure. Our research contributes to the understanding of the functioning of natural ecosystems, which can be useful for responsible forest management.

**Abstract:**

The study was conducted in the Białowieża Forest, which is the only place in Europe where the full development cycle of forests takes place on a large scale. The objective of this study was to compare spider assemblages inhabiting tree trunks and tree branches in the optimal, terminal and regeneration phases of a primeval oak–lime–hornbeam stand, in terms of their abundance, species diversity and species richness. Spiders of tree branches were sampled using a sweep net into which branches were shaken, while spiders inhabiting tree trunks were collected using traps made of corrugated cardboard placed around the trunks. The three analysed phases did not differ in terms of total species richness. We found that the species diversity of both foliage-dwelling and trunk-dwelling spider assemblages was higher in the terminal phase compared to other phases, which may indicate that the former phase offered the most diverse niches for spiders as a result of the significant disturbance in the stand structure. In addition, we found fewer spider individuals and species in individual samples collected on tree branches from a plot in the regeneration phase compared to the other phases, which may be a consequence of the structure of the stand in this phase (low canopy cover, lush herbaceous vegetation).

## 1. Introduction

Trees, because of their large size and complex structure, provide many unique and important microhabitats (e.g., trunks, foliage, branches, cavities) for many groups of invertebrates, including spiders [1,2,3,4]. Despite this fact, the spider fauna of trees is a rare subject of research. Blick [5] estimated the knowledge of spiders inhabiting tree trunks in forests of Central Europe at 5% compared to that of spiders inhabiting the ground. There are also few studies on spiders inhabiting tree branches [6]. Furthermore, in many of these studies, material was collected from different parts of trees or their strata and analysed together because of the use of nonselective methods such as insecticide fogging [7,8]. This may lead to incorrect conclusions, as individual microhabitats on trees vary greatly in structure and microclimatic conditions, and thus the spider assemblages inhabiting them are likely to be different. In contrast to such studies, here we separately analysed two microhabitats on trees, tree trunks and tree branches, in relation to the forest stand development phase.

The present study was conducted in the Białowieża National Park, where valuable natural European lowland forests are preserved. These forests are characterised as a multispecies community of trees, with a multi-layered and unevenly aged stand structure, considerable tree heights and a large amount of dead wood [9,10]. Unlike most forests in Europe, a complete cycle of forest stand development takes place here [11,12]. Several developmental phases can be distinguished in that cycle; however, their number is a matter of dispute. For example, Miścicki [13] defined eight phases (initial, juvenile, even-aged pole, premature, optimal, terminal, decay, regeneration), whereas Bobiec et al. [11] distinguished six phases (regeneration, young, pole, late pole, optimal and terminal). In our study, we included three of these phases, optimal, terminal/decay and regeneration, which are relatively easy to distinguish because of the significant differences in their stand structure. It is worth emphasising, however, that the decay (terminal) phase does not occur in most European forests as a result of logging and the elimination of dying trees.

Changes in invertebrate assemblages during the development cycle of temperate forests have rarely been studied, and when they have, the studies involved monocultures or forest plantations [14,15,16]. In the Białowieża Forest, such studies were conducted by Trojan et al. [17] in pine stands and included 27 taxa of animals (including spiders). Moreover, Stańska and Stański [18] studied plant-dwelling spider assemblages in different developmental phases of a primeval oak–lime–hornbeam stand. This study contained only spiders inhabiting herbaceous vegetation, which is a completely different habitat than trees. To our knowledge, there are no other studies discussing this problem in primeval forests.

Spiders are an excellent model group with which to study the effects of changes in the structure of a forest on the animal assemblages that inhabit it. Their abundance, species richness and diversity are affected by such factors as tree species diversity, the type of forest, its structure and canopy openness [19,20,21,22,23,24].

The objectives of this study were: (1) to determine the species composition of spider assemblages on tree branches and tree trunks in optimal, terminal and regeneration phases of primeval oak–lime–hornbeam forest; (2) to compare spider assemblages between these phases of stand development in terms of spider abundance (adults and juveniles separately), species richness and species diversity; and (3) to assess how the number of individuals and the number of species have changed over time (particular sampling months).

Many studies have shown that structurally diverse habitats support high species diversity, species richness and an abundance of spiders because they provide a large number of niches and diverse microhabitats [25,26,27,28]. Therefore, we hypothesised that spider species richness, diversity and abundance would be the highest on a plot with the terminal phase where, on the one hand, significant habitat disturbance has occurred (broken branches, emerging canopy gaps) and, on the other hand, mature, standing trees are still present.

## 2. Materials and Methods

### 2.1. Study Site

The Białowieża Forest, located on the Polish–Belarusian border, is a remnant of forests that covered much of temperate Europe centuries ago. Most of the area in the Polish part is under forest management, but the most valuable forest stands are protected as the Białowieża National Park (hereafter BNP). Human activity here is limited to scientific research and guided tourist walks. Forest stands in the BNP may be considered primeval forests, as evidenced by their multi-layered and uneven-aged structure, multispecies tree community, significant tree heights and a large amount of dead wood [9,10]. In addition, forest stands in the BNP have a heterogeneous structure, which is manifested in the fact that different developmental stages or forest types occupy small areas next to each other [11].

Our study was conducted in an oak–lime–hornbeam stand, which is the most common forest type in the BNP. In each of the three developmental phases of the forest, optimal, terminal and regeneration, one study plot (20 × 40 m rectangle) was selected. Trees growing on the optimal phase plot (52°43ʹ50” N; 23° 51ʹ40” E) were characterised by good vitality and a large diameter at breast height, and their crowns formed a dense canopy (above 90% cover). The most common tree species in this developmental phase were European hornbeam *Carpinus betulus*, pedunculate oak *Quercus robur*, Norway spruce *Picea abies*, small-leaved lime *Tilia cordata* and Norway maple *Acer platanoides*. Trees on the plot (52° 43ʹ30” N; 23°51ʹ50” E) with the forest stand in the terminal phase of development had a large diameter at breast height, but were usually in poor condition, as indicated by the presence of numerous dead branches and large fragments of decayed wood. Gaps in the canopy of the forest stand resulted from many large branches breaking off from the trunks (canopy cover of about 80%). The dominant tree species on this plot were European hornbeam, pedunculate oak and Norway spruce. The forest stand in the regeneration phase (52°43ʹ10” N; 23°51ʹ00” E) was characterised by the presence of patches without trees or with single trees as a result of strong winds that had felled most of the old trees 20 years before our research. Therefore, the canopy cover was very thin (about 20%), and lying deadwood was very abundant. In addition, there were a large number of young trees. The forest stand on this plot consisted mainly of European hornbeam, small-leaved lime, Norway spruce and pedunculate oak.

### 2.2. Data Collection

Spiders were collected from tree branches from April to November 2000. A total of ten samples were collected from each study plot: one sample in April, two samples in May, two samples in June, two samples in July, two samples in October and one sample in November. Spiders were collected from the branches of different trees, each time being selected randomly. The spiders belonged to different species, but the European hornbeam was sampled most frequently because this species had the easiest access to these branches (they were at the right height). On each sampling date, material was collected on each plot from ten branches of a similar size (1 × 0.5 m), located at a height of 1–2 m. The sampled branches were placed in the sweep net and then shaken vigorously, after which they were carefully inspected to collect spiders that had not fallen into the net. Because of the low abundance of spiders, the material from ten branches collected from each plot on each sampling date was combined into one sample.

Spiders on tree trunks were collected from June 1998 to October 2000 every month except November, December, January and February. Spiders were sampled using traps made of corrugated cardboard (25 cm wide), which were placed around trunks with their corrugated surface facing inwards. On each plot, five traps were placed on live trees (the same procedure was used throughout the study period) of a similar diameter (two on hornbeam, two on lime, one on spruce) at a height of 1.5 m above the ground. During sampling, the traps were removed from the trunks and the spiders sitting on them were collected. In addition, spiders that remained on the bark at a trap site were also collected. The material from five traps collected from each plot on each sampling date was pooled as one sample due to the low abundance of spiders. In total, the material was collected 18 times in the optimal and regeneration phase and 19 times in the terminal phase (during one control in the optimal phase and one control in the regeneration phase, some destroyed traps were found; thus, two samples were excluded from the analysis).

The collected spiders were preserved in 75% alcohol and then identified in the laboratory to the species level or, if this was not possible, as in the case of many juvenile specimens, to the higher taxon. The material was deposited at the Institute of Biological Sciences, Siedlce University of Natural Sciences and Humanities, Poland.

### 2.3. Statistical Analysis

To estimate sampling sufficiency on the study plots, richness estimators (Chao1, Chao2, Jackknife1, Jackknife2 and Michaelis–Menten) were calculated using 100 randomisations in EstimateS software version 9.1.0 [29]. To check whether the plots in different developmental phases differed in terms of species richness (i.e., the number of species recorded throughout the study period), rarefaction curves were calculated for the observed species richness with 95% confidence limits, based on the bootstrap method with 100 replications [30]. The species richness computed for each phase was considered significantly different when the confidence limits did not overlap [31,32].

The formula for the Shannon index (H’) was used to calculate the species diversity:

H’ = − Σ pi ln (pi)

where pi is the proportion of individuals of species i [33].

The Hutcheson test was used to compare Shannon diversity indices calculated for plots in different developmental phases using formulas prepared in Excel [34].

Generalised linear models (GLMs) were used to assess the association of the number of collected spider individuals and spider species with the developmental phase of the forest stand and the sampling period. In the models where the response variable was the number of collected spider species and the number of adult individuals, Gaussian error distribution and the identity link function were used. In the model where the response variable was the number of collected juvenile spider individuals, the Gaussian error distribution and the log-link function were used. The “developmental phase” and “sampling month” were treated as fixed categorical explanatory variables. If a given variable showed a significant effect in a model, paired contrasts were calculated to find significant differences between its levels. These calculations were performed in SPSS 21.0 for Windows.

## 3. Results

### 3.1. Spiders of Tree Branches

A total of 725 spider individuals from eight families were collected on tree branches during the study period (320 individuals in the optimal phase, 236 individuals in the terminal phase and 169 individuals in the regeneration phase). Juvenile spiders dominated in the collected material in each developmental phase (655 individuals in total, ca. 90%). A total of 591 individuals were identified to the species level: 266 from the optimal phase plot, 188 from the terminal phase plot and 137 from the regeneration phase plot. A total 24 species were identified (17 in the optimal phase, 16 in the terminal phase and 11 in the regeneration phase), of which 8 were common to all plots. A total of 13 species were represented by only 1 individual captured on a given plot (Table 1). However, the calculated estimators indicated much higher species richness, especially for the optimal phase plot and the terminal phase plot (Table 2).

**Figure 1:**
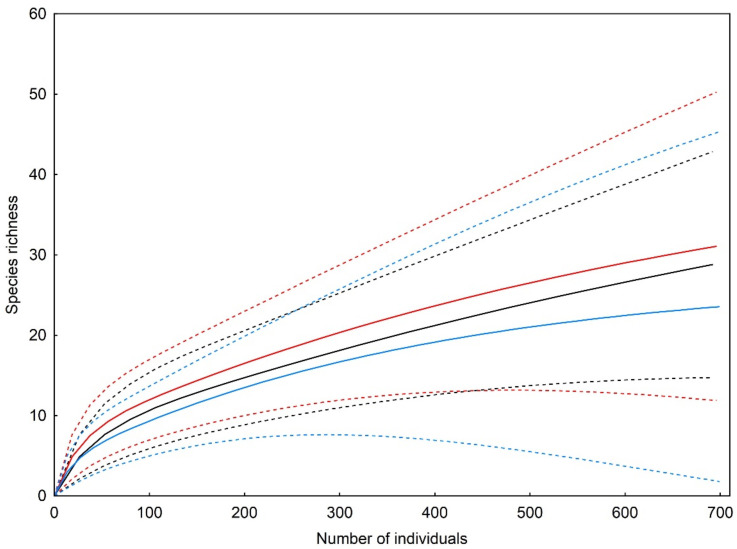
Individual-based rarefaction (solid) curves with 95% confidence limits (dashed curves) comparing species richness of branch-dwelling spider assemblages in three developmental phases of primeval oak–lime–hornbeam forest: the optimal phase (black), terminal phase (red) and regeneration phase (blue).

*Trematocephalus cristatus* was the most abundant species both in the optimal phase (where it accounted for 41.7% of the individuals identified to the species level) and the regeneration phase (37.2%), while *Neriene peltata* was most abundant in the terminal phase (25.5%), although the proportion of the former species was only slightly lower in this case (Table 1). The analysis of rarefaction curves revealed that the three studied developmental phases did not differ from each other in terms of the total species richness found on tree branches (Figure 1).

The highest species diversity of spider assemblages from tree branches was found in the terminal phase (H’ = 2.01), followed by the optimal phase (H’ = 1.91) and the regeneration phase of the forest stand development (H’ = 1.55). The Hutcheson test revealed differences between the optimal phase and the regeneration phase (t_296_ = 3.19; *p* = 0.002), as well as between the terminal phase and the regeneration phase (t_281_ = 4.10; *p* < 0.001), while no differences were found between the optimal phase and the terminal phase (t_438_ = 1.08; *p* = 0.283).

**Figure 2:**
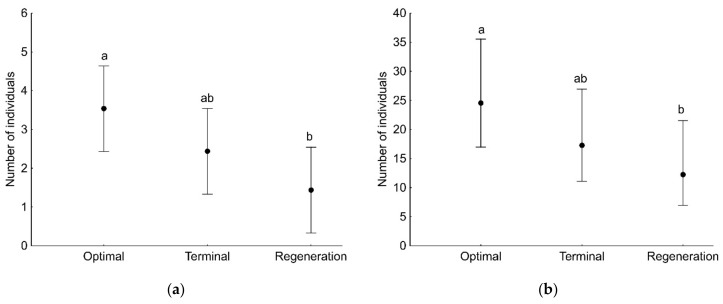
The number of adult (**a**) and juvenile (**b**) spider individuals (mean with 95% confidence limits) recorded on tree branches in a single sample in three developmental phases of primeval oak–lime–hornbeam forest. Different letters indicate significant differences between developmental phases.

**Figure 3:**
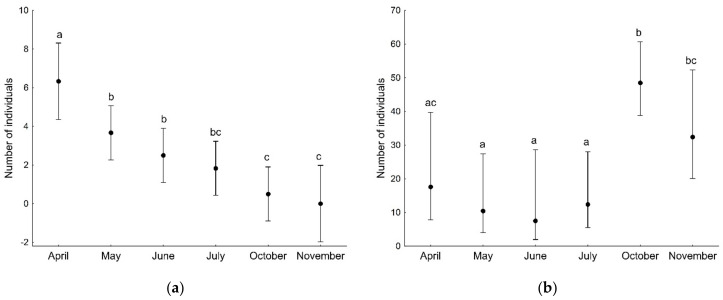
The number of adult (**a**) and juvenile (**b**) spider individuals (mean with 95% confidence limits) recorded on tree branches in particular sampling months. Different letters indicate significant differences between sampling months.

**Figure 4:**
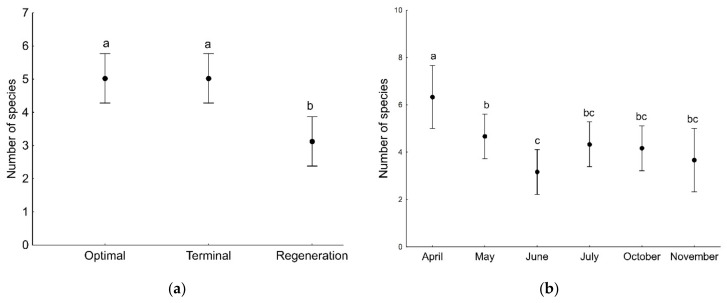
The number of spider species (mean with 95% confidence limits) recorded on tree branches in a single sample in three developmental phases of primeval oak–lime–hornbeam forest (**a**) and in particular sampling months (**b**). Different letters indicate significant differences between particular developmental phases (**a**) and particular sampling months (**b**).

GLMs showed that the number of both adult and juvenile spider individuals, as well as the number of species (found in a given sample), was associated with the developmental phase of the forest stand and the sampling period (Table 3). Significantly more adult and juvenile spider individuals were found on the plot with the optimal phase compared to the regeneration phase (Figure 2). The number of adult individuals captured on branches decreased during the study period, i.e., from April to November (Figure 3a), in contrast to the number of juveniles, which increased significantly in the last two sampling months, i.e., October and November (Figure 3b). The number of spider species (found in a given sample) was significantly lower on the plot in the regeneration phase compared to the other plots (Figure 4a). The number of species was significantly higher at the beginning of the study period (April) compared to the other months (Figure 4b).

### 3.2. Spiders of Tree Trunks

A total 2146 spider individuals belonging to 17 families were sampled on tree trunks during the study period (829 individuals in the optimal phase, 695 individuals in the terminal phase and 622 individuals in the regeneration phase). Juvenile spiders dominated in the collected material in each developmental phase (1845 individuals in total, ca. 86%). A total of 1610 individuals were identified to the species level: 621 from the optimal phase plot, 536 from the terminal phase plot and 453 from the regeneration phase plot. A total of 33 species were found (24 in the optimal phase, 23 in the terminal phase and 19 in the regeneration phase), of which 14 were common to all plots. A total of 13 species were represented by only 1 individual captured on a given plot (Table 4). The calculated estimators indicated higher species richness, especially for the terminal phase plot, where the sampling completeness was the lowest (Table 5). The most abundant spider species was *Anyphaena accentuata*, followed by *Amaurobius fenestralis*, in each phase of the stand development (Table 4).

**Figure 5:**
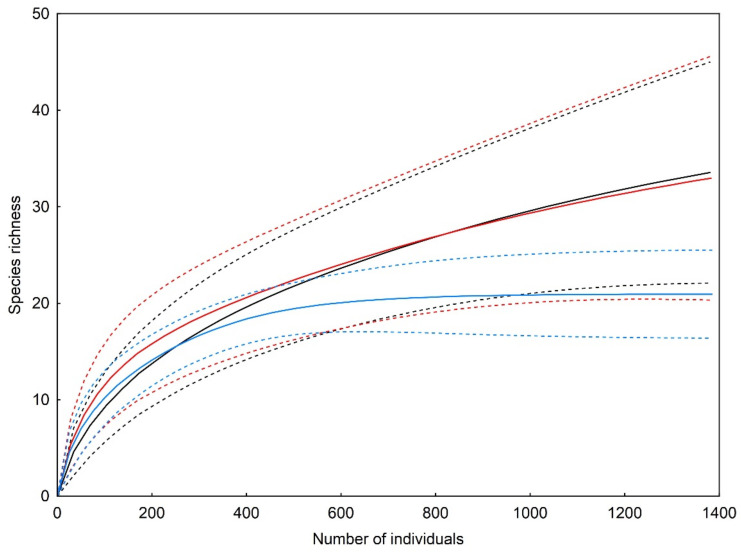
Individual-based rarefaction (solid) curves with 95% confidence limits (dashed curves) comparing species richness of trunk-dwelling spider assemblages in three developmental phases of primeval oak–lime–hornbeam forest: the optimal phase (black), terminal phase (red) and regeneration phase (blue).

The highest species diversity of spider assemblages from tree trunks was found in the terminal phase (H’ = 1.81), followed by the regeneration phase (H’ = 1.58) and the optimal phase of the forest stand development (H’ = 1.50). The Hutcheson test revealed differences between the optimal phase and the terminal phase (t_1148_ = 4.08; *p* < 0.001), as well as between the terminal phase and the regeneration phase (t_947_ = 2.85; *p* = 0.004), while no differences were found between the optimal phase and the regeneration phase (t_985_ = 0.94; *p* = 0.348). The analysis of rarefaction curves revealed that the three developmental phases did not differ from each other in terms of the total species richness of spider assemblages inhabiting tree trunks (Figure 5).

**Figure 6:**
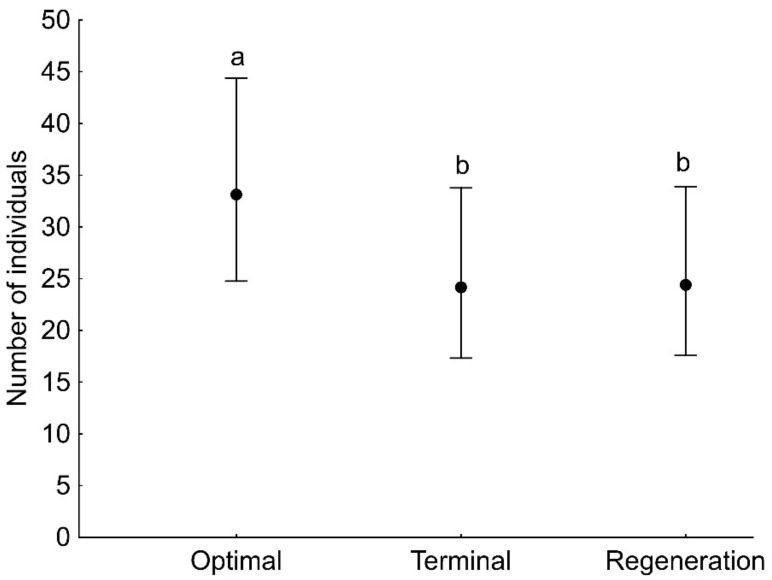
The number of juvenile spider individuals (mean with 95% confidence limits) recorded on tree trunks in a single sample in three developmental phases of primeval oak–lime–hornbeam forest. Different letters indicate significant differences between developmental phases.

**Figure 7:**
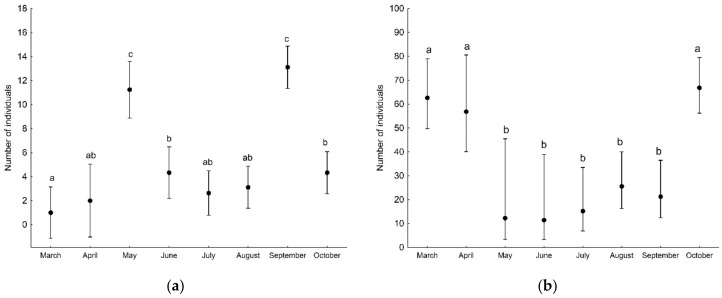
The number of adult (**a**) and juvenile (**b**) spider individuals (mean with 95% confidence limits) recorded on tree trunks in particular sampling months. Different letters indicate significant differences between sampling months.

The number of adult spider individuals and the number of species (found in each sample) were not associated with the developmental phase of the forest stand, while the number of juveniles was (Table 6). More juveniles were found in the optimal phase compared to the other two phases (Figure 6). Moreover, both the number of adults and juveniles were associated with the month of sampling, while number of species was not (Table 6). More adult individuals were captured in May and September (Figure 7a), while juveniles were significantly more numerous in March, April and October compared to the other months (Figure 7b).

## 4. Discussion

The hypothesis that the terminal phase, compared to the optimal and regeneration phases of stand development, would be characterised by higher spider abundance, species richness and species diversity was only confirmed for the last variable. We found the Shannon index was higher in the terminal phase compared to the other phases for both foliage-dwelling and trunk-dwelling spider assemblages. This fact may support our assumption that the most diverse niches for spiders exist in the terminal phase forest, as a result of significant disturbance in the stand structure. This disturbance was caused by the continuing process of old trees dying. As a result, some of the branches have broken off, and thus the crowns of the trees have become less dense. The structure of such a forest becomes very varied, with dead wood lying on the ground and lush herbaceous vegetation growing in places where gaps in the crowns have been created, and, at the same time, numerous trees of a large size provide varied niches for many groups of invertebrates. The phenomenon where significant variation in habitat structure translates into greater species diversity has been widely reported in the literature [25,35,36].

The present study showed that differences between the stand development phases do exist, but mainly indicated a lower number of spider individuals and number of species collected in individual samples on the plot in the regeneration phase compared to, above all, the plot in the optimal phase, where these variables reached the highest values. In our opinion, these differences may be explained, among other factors, by canopy cover, which was highest on the plot in the optimal phase and lower on the plot in the regeneration phase. The significant effect of canopy cover on spider assemblages in forests has been demonstrated by some authors. For example, Košulič et al. [20], studying epigeic spiders, showed that species richness was highest in places with medium canopy openness, while an open canopy supported the abundance of rare and threatened species. In addition, Oxbrough et al. [37] found that an open canopy favoured spider species typically absent in forest, and on a large scale, increased the abundance and species richness. In our study, we did not observe the occurrence of species associated with open habitats in the regeneration phase, but this may be due to the structure of the oak–lime–hornbeam forest in the BNP manifested by the fact that different developmental phases occupy relatively small areas located close to each other [11]. On the other hand, the canopy openness translated into the degree of herbaceous vegetation development in our study [18], and this may explain the differences between particular phases in abundance and species richness, at least for foliage-living spiders.

A higher number of juvenile and adult spiders in individual samples was collected on tree branches in the optimal phase compared to the regeneration phase, which may have resulted from poor herbaceous vegetation cover in the former phase [18]. The foliage can provide a kind of substitute for herbaceous vegetation, especially in the case of low-lying branches, and therefore, where herbaceous vegetation is less developed, spiders may be more likely to inhabit tree leaves. On the other hand, Stenchly et al. [38] found that the abundance and species richness of spiders from different strata, including those collected in tree crowns, were positively affected by herbaceous cover.

The sampled branches were located at a similar height from the ground as many herbaceous plants and to some extent resembled them in structure. This allowed us to assume that the fauna of spiders living on branches, at least those located not too high off the ground, should largely consist of plant-dwelling species. Our study largely confirmed this assumption. For example, *Trematocephalus cristatus*, the most abundant species on plots in the optimal and regeneration phases, was also abundant on herbaceous vegetation [18]. Other species, such as *Cyclosa conica*, *Enoplognatha ovata* and *Diaea dorsata*, were also collected both from tree branches and herbaceous vegetation in the study plots [18]. On the other hand, species such as *Linyphia triangularis* or *Bathyphantes nigrinus*, which were collected in large numbers on herbaceous vegetation, were not found on tree branches or only in small numbers. In addition, the fauna of foliage spiders should also include species living on tree trunks, as they can reach the leaves relatively easily. We found that *Anyphaena accentuata*, the most abundant spider on tree trunks, was also abundant on foliage, and was additionally collected on herbaceous vegetation [18]. However, the fact that more than half of the species found on branches were represented by only one individual on a given plot suggests that many species may have ended up there by chance and this is not their preferred habitat.

Only three species from the spider assemblages on tree trunks contributed 5% or more in each plot. Two of them, i.e., *Anyphaena accentuata* and *Amaurobius fenestralis*, clearly dominated, together accounting for 70–80% (dependent on plot) of all individuals identified to the species level. In addition, these species were collected in every (*Amaurobius fenestralis*) or almost every month (*Anyphaena accentuata*) of the trapping period. This showed that tree trunks are a common habitat for them, even though they may also live in others [5,18,39,40]. Furthermore, among the captured species, we can also include *Segestria senoculata* [40] and *Neriene montana* as typical inhabitants of tree trunks [23,41,42]. Other species were captured on tree trunks only in single months or in small numbers (more than 1/3 of the species were only represented on a given plot by a single captured individual). This shows that tree trunks serve as an incidental or temporary habitat for them, providing shelter or prey [43]. In addition, the fact that some species were recorded only in March, when winter still prevails in the Białowieża Forest, and/or in October when winter is approaching, may suggest that tree trunks are a wintering site for them. For example, *Diaea dorsata* was collected on tree trunks only in March, April and October, while it was found mainly on leaves from April to July. This may indicate that tree trunks are a substitute habitat for this species in the period when leaves have not yet appeared on trees and herbaceous vegetation has not fully developed, or that this is its overwintering site.

Spiders of tree crowns in the Białowieża Forest, outside primeval stands, were studied by Otto and Floren [7], who applied insecticidal knockdown fogging. They found the most abundant species were *Diaea dorsata* (21.8%), *Anyphaena accentuata* (16.1%), *Enoplognatha ovata* (13.5%) and *Paidiscura pallens* (9.9%). Of these species, we also found the first three in significant numbers on branches, although their proportions were different, while only *Anyphaena accentuata* was abundant on tree trunks. On the other hand, Otto and Floren [7] did not find *Amaurobius fenestralis* at all, which we found in large numbers on tree trunks. This may suggest that the fauna of spiders in tree crowns differs from that in tree trunks, whereas the fauna of spiders collected on branches is similar regardless of the height of the branches above the ground.

We found that the number of individuals, both adults and juveniles, and spider species (only in the case of foliage-living spiders) varied between the months evaluated in our study. This is certainly due to the phenology of individual spider species and changes in some habitat parameters (e.g., humidity, temperature) during the sampling period, although these were unfortunately not measured throughout the study period.

## 5. Conclusions

The analysed developmental phases of the oak–lime–hornbeam stand did not differ in terms of the total spider species richness. However, we found that species diversity of both foliage-dwelling and trunk-dwelling spider assemblages was higher in the terminal phase compared to other phases, which may indicate that this phase offers the most diverse niches for spiders due to the significant disturbance in the forest stand structure. The fauna of spiders inhabiting tree branches consisted largely of plant-dwelling species. We found that the fauna of tree trunks on each plot was dominated by two species—*Anyphaena accentuata* and *Amaurobius fenestralis*. For most spider species, tree trunks and branches are only temporary habitats or places where they can hide or overwinter.

## Figures and Tables

**Table 1:** Spiders collected on tree branches in three developmental phases of oak–lime–hornbeam stands in the Białowieża National Park (spider families, genus and species in alphabetical order). The percentages presented in parentheses, next to the number of individuals, show the proportion of each species. All individuals identified to the species level were included in the percentage composition, but values are only shown for species that reached at least 5%. Abbreviations: Ad./Juv.— number of adult/juvenile spider individuals, un.—individuals identified only to the family level. Roman letters indicate the months in which a given species was recorded.

Family/Genus/Species	Optimal Phase		Terminal Phase		Regeneration Phase	
	Ad./Juv.	Months	Ad./Juv.	Months	Ad./Juv.	Months
Anyphaenidae						
*Anyphaena accentuata*	(12%) -/32	IV–VII, X, XI	(20%) 1/37	IV–VII, X, XI	(36%) 1/49	IV–VII, X, XI
Araneidae						
*Araneus diadematus*			-/1	VII		
*Araniella* sp.	-/1				-/5	
*Cyclosa conica*	4/5	IV, V, VII, X	1/7	IV, V, X, XI	2/-	IV
*Clubionidae*						
*Clubiona* sp.	-/2				-/2	
Linyphidae						
*Diplocephalus picinus*			1/-	VI		
*Entelecara acuminata*	1/-	VII	1/-	VII		
*Helophora insignis*	-/5	VII	-/2	VII		
*Linyphia triangularis*	1/-	VII				
*Linyphiidae un.*	-/6		-/12		-/3	
*Neriene clathrata*	-/1	VII				
*Neriene emphana*	(6%) 2/15	IV, VI, VII	2/7	IV–VII, XI	-/1	V
*Neriene montana*	-/1	X	-/4	VII, X	1/-	V
*Neriene peltata*	(6%) 9/7	IV, V, XI	(26%) 6/42	IV–VI, X, XI	(8%) -/11	X
*Neriene* sp.	-/20		-/3		-/1	
*Pityohyphantes phrygianus*			-/1	X		
*Porrhomma pygmaeum*	5/-	IV			3/-	V
*Tapinocyba insecta*	1/-	IV				
*Tapinocyba pallens*			1/-	IV		
*Trematocephalus cristatus*	(42%) -/111	V, X, XI	(22%) -/42	X, XI	(37%) 1/50	IV, V, X, XI
Philodromidae						
*Philodromus dispar*	1/-	V				
*Philodromus* sp.	-/7		-/6		-/1	
Tetragnathidae						
*Metellina* sp.	-/2		-/4		-/5	
*Metellina mengei*	2/-	X	1/-	X		
*Tetragnatha montana*					1/-	V
*Tetragnatha* sp.	-/8		-/9		-/7	
Theridiidae						
*Enoplognatha ovata*	(9%) 6/19	V, VI	(9%) 7/10	V–VII	(8%) 3/8	V–VII
*Robertus scoticus*	1/-	IV				
*Theridiidae un.*			-/1			
*Theridion* sp.	-/7		-/12		-/7	
*Theridion varians*					1/-	VII
Thomisidae						
*Diaea dorsata*	(14%) 1/36	IV–VII, X	(7%) 1/12	IV–VII, XI	(4%) -/5	IV–VII
*Ozyptila* sp.					-/1	
*Xysticus lanio*			1/-	V		
*Xysticus* sp.	-/1		-/1			
Total no. of individuals	34/286		23/213		13/156	

**Table 2:** Observed species richness and species richness estimates for spider assemblages from tree branches in three developmental phases of oak–lime–hornbeam stand. Sampling completeness was calculated using Chao1 estimator.

	Optimal Phase	Terminal Phase	Regeneration Phase
Observed richness	17	16	11
Estimates			
Chao1 ± SD	41 ± 31	40 ± 31	19 ± 12
Chao2 ± SD	53 ± 44	45 ± 36	27 ± 21
Jackknife1 ± SD	25 ± 3	23 ± 2	16 ± 2
Jackknife2	32	29	20
Michaelis–Menten	22	20	15
Sampling completeness	41%	40%	58%

**Table 3:** Results of generalised linear models assessing the effect of the developmental phase of the tree stand and sampling month on the abundance and the species richness of spider assemblages of tree branches.

Effect	Wald χ^2^	df	*p*
**Abundance of adult individuals**			
Intercept	53.95	1	<0.001
Developmental phase	7.21	2	0.027
Sampling month	31.66	5	<0.001
**Abundance of juvenile individuals**			
Intercept	235.53	1	<0.001
Developmental phase	7.91	2	0.019
Sampling month	29.18	5	<0.001
**Species richness**			
Intercept	375.06	1	<0.001
Developmental phase	17.36	2	<0.001
Sampling month	16.01	5	0.007

**Table 4:** Spiders collected on tree trunks in three developmental phases of oak–lime–hornbeam stands in the Białowieża National Park (spider families, genus and species in alphabetical order). The percentages presented in parentheses, next to the number of individuals, show the proportion of each species. All individuals identified to the species level were included in the percentage composition, but values are only shown for species that reached at least 5%. Abbreviations: Ad./Juv.—number of adult/juvenile spider individuals, un.—individuals identified only to the family level. Roman letters indicate the months in which a given species was recorded.

Family/Genus/Species	Optimal Phase		Terminal Phase		Regeneration Phase	
	Ad./Juv.	Months	Ad./Juv.	Months	Ad./Juv.	Months
Agelenidae						
Agelenidae un.	-/1					
*Coelotes atropos*	5/-	VIII, IX	5/6	IV, VI–IX	2/-	VII, IX
Amaurobiidae						
*Amaurobius fenestralis*	(32%) 52/146	III–X	(31%) 62/106	III–X	(25%) 38/74	III–X
Anyphaenidae						
*Anyphaena accentuata*	(49%) 4/299	III–V, VII–X	(38%) 6/197	III–V, VII–X	(52%) 7/227	III–VI, VIII–X
Araneidae						
Araneidae un.					-/1	
*Cyclosa conica*	-/2	IV				
*Nuctenea umbratica*					2/-	VIII, IX
Clubionidae						
*Clubiona caerulescens*	1/-	V				
*Clubiona lutescens*	3/-	VII, IX	2/-	VII, VIII	4/-	VII, IX
*Clubiona* sp.	-/110		-/33		-/4	
*Clubiona subsultans*	1/-	X	3/-	X		
Dictynidae						
*Dictyna* sp.					-/1	
Gnaphosidae						
*Haplodrassus cognatus*	1/-	III	1/-	VI	1/-	V
*Haplodrassus* sp.	-/24		-/48		-/44	
Linyphiidae						
*Agyneta ramosa*			1/-	VI		
*Drapetisca socialis*	18/7	VI–X	11/13	VI–X	13/3	V–IX
*Helophora insignis*	1/-	VIII	1/-	VIII		
*Labulla thoracica*	1/5	VI–VIII	-/3	VII, VIII	1/-	IX
*Lepthyphantes minutus*	2/-	VIII, IX	7/1	VII–IX		
*Lepthyphantes* sp.	-/7					
*Linyphiidae un.*	-/15		-/22		-/39	
*Lophomma punctatum*	1/-	III				
*Neriene clathrata*					1/-	VI
*Neriene montana*	-/4	III, IV, VIII, X	-/15	IV, VIII–X	2/8	III, V, VI, VIII, X
*Neriene* sp.	-/2					
*Savignia frontata*			1/-	X		
*Trematocephalus cristatus*	-/11	III, IV, X			-/4	III
Lycosidae						
*Piratula hygrophila*					1/-	IX
Mimetidae						
*Ero furcata*			1/-	V		
Philodromidae						
*Philodromus* sp.	-/11		-/19		-/13	
Pisauridae						
*Dolomedes fimbriatus*	-/1	X				
Salticidae						
*Neon reticulatus*			3/-	V, VII		
*Neon* sp.			-/1			
*Salticidae un.*			-/1		-/1	
Segestriidae						
*Segestria senoculata*	1/10	V, VIII–X	(9%) 5/43	V–X	(6%) 3/24	V–X
Tetragnathidae						
*Metellina merianae*			1/-	VII		
*Tetragnatha* sp.	-/2		-/1		-/2	
Theridiidae						
*Dipoena nigroreticulata*			1/-	VI	1/1	III, X
*Enoplognatha ovata*	2/1	VI	3/3	V–VIII	1/4	VI, IX
*Steatoda bipunctata*	1/2	VI, IX	2/11	V–IX	3/15	III, VII–X
*Theridion mystaceum*	1/-	V	3/-	V, VI	3/-	V–VII
*Theridion* sp.	-/28		-/34		-/58	
*Platnickina tincta*	1/2	VII, X	-/1	IV	2/1	VI, IX
*Theridion varians*	-/2	III				
Thomisidae						
*Diaea dorsata*	(5%) -/32	III, IV, X	-/18	III, IV, X	-/7	III, IV, X
*Ozyptila praticola*	1/-	VIII				
*Ozyptila* sp.	-/5				-/3	
*Xysticus* sp.	-/3				-/3	
Total no. of individuals	97/732		119/576		85/537	

**Table 5:** Observed species richness and species richness estimates for spider assemblages of tree trunks in three developmental phases of oak–lime–hornbeam stand. Sampling completeness was calculated using Chao1 estimator.

	Optimal Phase	Terminal Phase	Regeneration Phase
Observed richness	24	23	19
Estimates			
Chao1 ± SD	35 ± 10	55 ± 40	22 ± 3
Chao2 ± SD	43 ± 16	42 ± 19	21 ± 2
Jackknife1 ± SD	34 ± 4	32 ± 3	24 ± 2
Jackknife2	42	38	23
Michaelis–Menten	33	27	23
Sampling completeness	69%	42%	86%

**Table 6:** Results of generalised linear models assessing the effect of the developmental phase of the tree stand and sampling month on the abundance and the species richness of spider assemblages of tree trunks.

Effect	Wald χ^2^	df	*p*
**Abundance of adult individuals**			
Intercept	183.62	1	<0.001
Developmental phase	3.84	2	0.146
Sampling month	138.80	7	<0.001
**Abundance of juvenile individuals**			
Intercept	583.29	1	<0.001
Developmental phase	6.81	2	0.033
Sampling month	51.06	7	<0.001
**Species richness**			
Intercept	362.97	1	<0.001
Developmental phase	2.14	2	0.342
Sampling month	4.11	7	0.767

## Data Availability

The data presented in this study are available on reasonable request from the corresponding author.
